# Anticholinesterase and Antioxidative Properties of Aqueous Extract of *Cola acuminata* Seed *In Vitro*

**DOI:** 10.1155/2014/498629

**Published:** 2014-11-18

**Authors:** Ganiyu Oboh, Ayodele J. Akinyemi, Olasunkanmi S. Omojokun, Idowu S. Oyeleye

**Affiliations:** ^1^Functional Foods and Nutraceuticals Unit, Department of Biochemistry, Federal University of Technology, PMB 704, Akure 340001, Nigeria; ^2^Department of Biochemistry, Afe Babalola University, PMB 5454, Ado Ekiti, Nigeria

## Abstract

*Background. Cola acuminata* seed, a commonly used stimulant in Nigeria, has been reportedly used for the management of neurodegenerative diseases in folklore without scientific basis. This study sought to investigate the anticholinesterase and antioxidant properties of aqueous extracts from* C. acuminata* seed* in vitro*.* Methodology.* The aqueous extract of* C. acuminata* seed was prepared (w/v) and its effect on acetylcholinesterase (AChE) and butyrylcholinesterase activities, as well as some prooxidant (FeSO_4_, sodium nitroprusside (SNP), and quinolinic acid (QA)) induced lipid peroxidation in rat brain* in vitro*, was investigated.* Results.* The results revealed that* C. acuminata* seed extract inhibited AChE (IC_50_ = 14.6 *μ*g/mL) and BChE (IC_50_ = 96.2 *μ*g/mL) activities in a dose-dependent manner. Furthermore, incubation of rat's brain homogenates with some prooxidants caused a significant increase *P* < 0.05 in the brain malondialdehyde (MDA) content and inhibited MDA production dose-dependently and also exhibited further antioxidant properties as typified by their high radicals scavenging and Fe^2+^ chelating abilities.* Conclusion.* Inhibition of AChE and BChE activities has been the primary treatment method for mild Alzheimer's disease (AD). Therefore, one possible mechanism through which the seed exerts its neuroprotective properties is by inhibiting cholinesterase activities as well as preventing oxidative-stress-induced neurodegeneration. However, this is a preliminary study with possible physiological implications.

## 1. Introduction 

Alzheimer's disease (AD), first described by the German neurologist Alois Alzheimer, is a neurodegenerative disease affecting the brain, which is an irreversible, progressive brain disease that slowly destroys memory and thinking skills and eventually even the ability to carry out the simplest tasks [1]. In recent years, studies have implicated oxidative stress to play a crucial role in neurodegenerative diseases such as Alzheimer's disease via lipid peroxidation of cell membrane of the neurons [2]. Of particular importance, the brain is an organ extremely susceptible to free radical damage because of its high consumption of oxygen and its relatively low concentration of antioxidant enzymes and free radicals scavengers. In most people, AD symptoms become visible usually after age 60. AD sufferers generally have a reduced amount of acetylcholine in their brain which accounts for the cholinergic dysfunction which is associated with the disease. Nowadays, the most prescribed drug class in pharmacotherapy of AD is the cholinesterase inhibitors (ChEIs) that block the breakdown of ACh [3]. Cholinesterases belong to a family of proteins that is widely distributed throughout the body in both neuronal and nonneuronal tissues and is classified as either acetylcholinesterase (AChE) or butyrylcholinesterase (BuChE) based on their substrate and inhibitor specificity [4].

Relevantly, production of free radicals and oxidative stress metal accumulation such as iron, copper, and zinc in the beta-amyloid plaques formed in the brains of AD patients has been claimed strongly to be associated with cognitive impairment in negative manner [5]. Therefore, it is more substantial for a drug candidate for treatment of AD to possess antioxidant activity besides cholinesterase inhibition.

Although the etiology of Alzheimer's disease (AD) is not fully understood, nevertheless, inhibition of acetylcholinesterase (AChE) and butyrylcholinesterase (BChE) activity has been accepted as an effective treatment/management strategy against mild AD [6, 7]. AChE inhibitors such as tacrine, donepezil, and rivastigmine are commonly used synthetic drugs for the treatment of Alzheimer's disease; however, these drugs are limited in use due to their adverse side effects. More recently, studies have shown that BChE is found in significantly higher quantities in AD plaques than in the plaques of age related nondemented brains [8]. However, most of the drug AChE inhibitors discovered do not alter BChE activity which is very critical to managing AD. Hence, recent efforts have focused on plant phytochemicals as natural sources of effective AChE and BChE inhibitors with little or no side effects which could be used as dietary intervention in the management of this disease.

**Figure 1:**
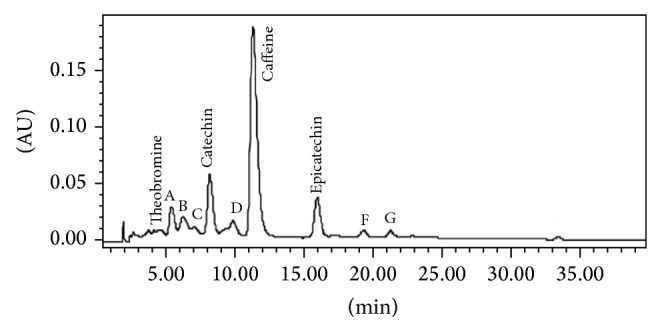
HPLC chromatograms of polyphenols and alkaloids in* Cola acuminata* seeds. Separation of polyphenols was performed on a LiChroCART 250-4 octadecylsilyl (ODS) C18, 5 *μ*m particle (RP-18 (5 *μ*m)) column (Merck) at 26°C. The guard column consisted of a LiChroCART 4-4 LiChrospher 100 RP-18 (5 *μ*m) (Merck). The binary mobile phase consisted of 2% acetic acid in water (A) and acetonitrile-water-concentrated acetic acid mixture (4 : 9 : 1 v/v/v) (B). Letters or numbers over the peak indicate unidentified polyphenolic compounds. (Source: [9]).

*Cola* is a tropical African genus which belongs to the Sterculiaceae family. The genus comprises about 140 species and the most commonly consumed is* Cola acuminata* (Pal. de Beauv) (Russel [10]).* Cola acuminata* is a bitter brown seed found in the pod of evergreen trees that are native to Africa. It has a strong cultural significance in West Africa, where, without these seeds, traditional hospitality and cultural and social ceremonies are considered incomplete. In Europe, America, and Nigeria, the seeds are used in the production of several pharmaceutical drugs, wines, and liquors [11–13]. The plant was introduced to the Central and South American countries where it became popular during the Slave Trade of the 17th century. This popularity resulted from its reputation as a stimulant, increasing energy and strength, dispelling drowsiness, and staving off hunger [14]. In traditional medicine, it is used in the management/treatment of memory loss and other neurodegenerative conditions. Niemenak et al., 2008 [9], reported that caffeine and theobromine were the major purine alkaloids in* Cola acuminata* seeds while catechin and epicatechin were the predominant polyphenols. The HPLC chromatogram of polyphenols and alkaloids in* Cola acuminata* is presented in Figure 1 as reported by Niemenak et al., 2008 [9]. However, based on the continuous search for natural products that are cholinesterase inhibitors and also due to the fact that* Cola acuminata* is used in folk medicine for memory-improvement till date, it is therefore expedient to assess its anticholinesterase activity as well as effect on some prooxidant (FeSO_4_, sodium nitroprusside, and quinolinic acid) induced oxidative stress in rats brain* in vitro*.

## 2. Materials and Methods

### 2.1. Sample Collection

Fresh samples of kola nut (*Cola acuminata*) seeds were purchased at the Erekesan market in Akure metropolis, Nigeria. Authentication of the samples was carried out at the Department of Biology, Federal University of Technology, Akure, Nigeria.

### 2.2. Chemicals and Reagents

All chemicals used were sourced from Sigma Co. (St. Louis, MO). Except if stated otherwise, all the chemicals and reagents used are of analytical grade, while the water used was glass distilled.

### 2.3. Aqueous Extract Preparation

The kola nut seeds were thoroughly washed in distilled water to remove any dirt, chopped into small pieces by table knife, air-dried, and milled into fine powder. The aqueous extracts of the seed were prepared by soaking 5 g of the grinded samples in 100 mL of distilled water for 24 hrs at 37°C. The mixture was later filtered through Whatman number 2 filter paper and centrifuged at 4000 rpm to obtain a clear supernatant which was then stored in the refrigerator for subsequent analysis [15].

### 2.4. *In Vitro* Anticholinesterase Assays

Inhibition of AChE was assessed by a modified colorimetric method of Perry et al. (2001) [16]. The AChE activity was determined in a reaction mixture containing 200 *μ*L of a solution of AChE (0.415 U/mL in 0.1 M phosphate buffer, pH 8.0), 100 *μ*L of a solution of 5,5′-dithio-bis(2-nitrobenzoic) acid (3.3 mM in 0.1 M phosphate-buffered solution, pH 7.0) containing NaHCO_3_ (6 mM), extract dilutions (0 to 100 *μ*L), and 500 *μ*L of phosphate buffer, pH 8.0. After incubation for 20 min at 25°C, acetylthiocholine iodide (100 *μ*L of 0.05 mM solution) was added as the substrate, and AChE activity was determined with an ultraviolet spectrophotometer from the absorbance changes at 412 nm for 3.0 min at 25°C. 100 *μ*L of butyrylthiocholine iodide was used as a substrate to assay butyrylcholinesterase enzyme, while all the other reagents and conditions were the same. The AChE and BChE inhibitory activities were expressed as percentage inhibition.

### 2.5. Lipid Peroxidation and Thiobarbituric Acid Reactions

The lipid peroxidation assay was carried out using the modified method of Ohkawa et al. [17]. 100 mL S1 fraction was mixed with a reaction mixture containing 30 mL of 0.1 M pH 7.4 Tris-HCl buffer, extract (0–100 mL), and 30 mL of 70 mM freshly prepared sodium nitroprusside. The volume was made up to 300 mL by water before incubation at 37°C for 1 h. The colour reaction was developed by adding 300 mL 8.1% SDS (sodium dodecyl sulphate) to the reaction mixture containing S1; this was subsequently followed by the addition of 600 mL of acetic acid/HCl (pH 3.4) mixture and 600 mL 0.8% TBA (thiobarbituric acid). This mixture was incubated at 100°C for 1 h. TBARS (thiobarbituric acid reactive species) produced were measured at 532 nm and the absorbance was compared with that of standard curve using MDA (malondialdehyde).

### 2.6. ABTS Radical Scavenging Ability

The ABTS radical (ABTS^∙^) (2,2′-azino-bis(3-ethylbenzthiazoline-6-sulphonic acid)) was generated by reacting an ABTS aqueous solution (7 mmol/L) with K_2_S_2_O_8_ (2.45 mmol/L, final concentration) in the dark for 16 h and adjusting the Abs734 nm to 0.700 with ethanol. 0.2 mL of appropriate dilution of the extract was added to 2.0 mL ABTS^∙^ solution and the absorbance was measured at 734 nm after 15 minutes. The trolox equivalent antioxidant capacity was subsequently calculated [18].

### 2.7. Fenton Reaction

The extract (0–100 *μ*L) was added to a reaction mixture containing 120 *μ*L of 20 mM deoxyribose, 400 *μ*L of 0.1 M phosphate buffer, and 40 *μ*L of 500 *μ*M of FeSO_4_, and the volume was made up to 800 *μ*L with distilled water. The reaction mixture was incubated at 37°C for 30 minutes and the reaction was then stopped by the addition of 0.5 mL of 2.8% trichloroacetic acid. This was followed by addition of 0.4 mL of 0.6% thiobarbituric acid (TBA) solution. The tubes were subsequently incubated in boiling water for 20 minutes. The absorbance was measured at 532. The OH^∙^ scavenging ability was subsequently calculated [19].

### 2.8. DPPH Free Radical Scavenging Ability

The free radical scavenging ability of the extracts against DPPH (1,1-diphenyl-2 picrylhydrazyl) free radical was evaluated as described by Gyamfi et al. (1999) [20]. Briefly, appropriate dilution of the extracts (0–500 *μ*L) was mixed with 1 mL, 0.4 mM methanolic solution containing DPPH radicals; the mixture was left in the dark for 30 min and the absorbance was taken at 516 nm. The DPPH free radical scavenging ability was subsequently calculated.

### 2.9. Fe^2+^ Chelation Assay

The Fe^2+^ chelating ability of the extract was determined using a modified method of Minotti and Aust [21], with a slight modification by Puntel et al. [22]. Freshly prepared 500 *μ*M FeSO_4_ (150 *μ*L) was added to a reaction mixture containing 168 *μ*L 0.1 M Tris-HCl (pH 7.4), 218 *μ*L saline, and the extracts (0–25 *μ*L). The reaction mixture was incubated for 5 min, before the addition of 13 *μ*L 0.25% 1,10-phenanthroline (w/v). The absorbance was subsequently measured at 510 nm. The Fe (II) chelating ability was subsequently calculated.

### 2.10. Determination of Total Phenol Content

The total phenol content was determined by mixing 0.2 mL of the sample extract with 2.5 mL 10% Folin-Ciocalteu reagent (v/v) and 2.0 mL of 7.5% sodium carbonate was subsequently added. The reaction mixture was incubated at 45°C for 40 min, and the absorbance was measured at 765 nm using a spectrophotometer. Gallic acid was used as standard while the total phenol content was subsequently calculated as gallic acid equivalent [23].

### 2.11. Determination of Total Flavonoid Content

The total flavonoid content was determined by mixing 0.5 mL of appropriately diluted sample with 0.5 mL methanol, 50 *μ*L 10% A1C1_3_, 50 *μ*L 1 M potassium acetate, and 1.4 mL distilled water and allowed to incubate at room temperature for 30 min. The absorbance of the reaction mixture was subsequently measured at 415 nm; quercetin is used as standard flavonoid. The total flavonoid content was subsequently calculated as quercetin equivalent. The nonflavonoid polyphenols were taken as the difference between the total phenol and total flavonoid content [24].

### 2.12. Data Analysis

The results of replicate experiments were pooled and expressed as mean ± standard deviation (SD) [25]. A one-way analysis of variance (ANOVA) was used to analyze the mean and the post hoc treatment was performed using Duncan multiple range test. Significance was accepted at *P* < 0.05. The EC_50_ (extract concentration causing 50% enzyme inhibition/antioxidant activity) was performed using nonlinear regression analysis.

## 3. Results

**Figure 2:**
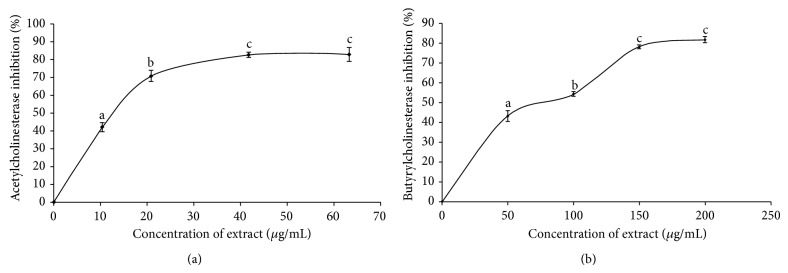
Anticholinesterase inhibitory activity, (a) acetylcholinesterase inhibitory activity and (b) butyrylcholinesterase inhibitory activity, of aqueous extract of* Cola acuminata*. Values represent means ± standard deviation of triplicate readings. Different letters above each concentration indicate significant differences (*P* < 0.05).

The AChE inhibitory potential of kola nut seed extract was investigated and the result is shown in Figure 2(a); the result revealed that the extract inhibited AChE activity in a dose-dependent manner (0–63.3 *μ*g/mL), having an IC_50_ (extract concentration causing 50% inhibition) value = 14.6 *μ*g/mL as presented in Table 1. Also, the ability of the extract to inhibit BChE activity* in vitro* was also investigated, and the result is presented in Figure 2(b). The result revealed that the extract inhibited BChE in a dose-dependent manner (0–200 *μ*g/mL) having an IC_50_ (extract concentration causing 50% inhibition) value = 96.2 *μ*g/mL as presented in Table 1.

**Figure 3:**
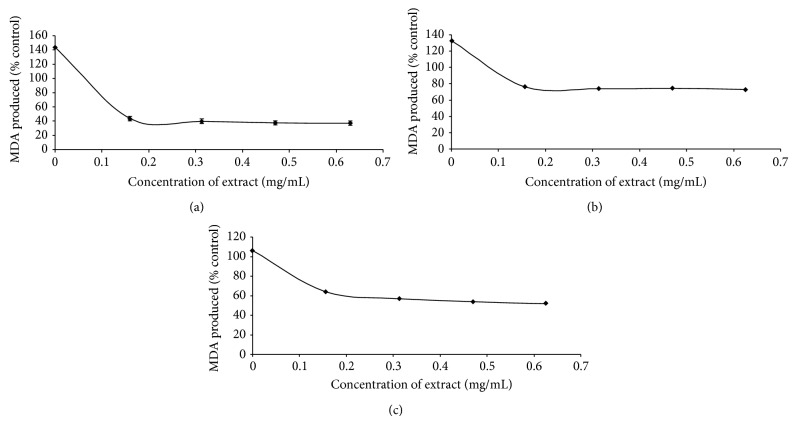
Inhibition of some prooxidants, (a) Fe^2+^, (b) sodium nitroprusside, and (c) quinolinic acid, induced lipid peroxidation in rat whole brain by aqueous extract of* Cola acuminata*. Values represent means ± standard deviation of triplicate readings.

**Figure 4:**
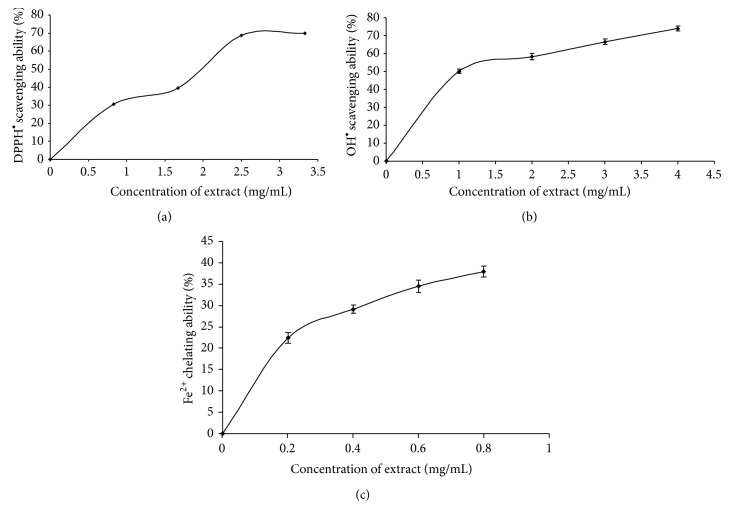
Some antioxidant parameters: (a) DPPH free radical scavenging, (b) OH radical scavenging, and (c) Fe^2+^ chelating abilities of aqueous extract of* Cola acuminata*. Values represent means ± standard deviation of triplicate readings.

Furthermore, incubation of the rat brain homogenate with some prooxidants caused a significant increase in the MDA production as presented in Figures 3(a)–3(c), respectively. However, the introduction of the extract inhibited MDA production in a dose-dependent manner (0.16–0.63 mg/mL). The ABTS radical (ABTS^∙^) scavenging ability presented as trolox equivalent antioxidant capacity is presented in Table 2. The result revealed that the extract scavenged ABTS^∙^ (2.65 mmol*·*TEAC/100 g). Also, the extract scavenged DPPH radical and OH radical and exhibited Fe^2+^ chelating activity in a dose-dependent manner as shown in Figures 4(a)–4(c). Furthermore, the total phenol (2.78 mg*·*GAE/g) and flavonoid (1.75 mg*·*QUE/g) contents of the nut seeds are presented in Table 2.

## 4. Discussion

Inhibition of acetylcholinesterase is considered as a promising approach for the treatment of Alzheimer's disease (AD) and for possible therapeutic applications in the treatment of Parkinson's disease, ageing, and myasthenia gravis [26, 27]. Meanwhile, BChE has been considered to be directly associated with the side effects of the AChE inhibitors and the existing drugs of Alzheimer's disease [28]. More recent studies have shown that BChE is found in significantly higher quantities in AD plaques than in the plaques of age related nondemented brains. Other recent studies have also reported that the unfavorable side effects profile of AChE inhibitors is not associated with their poor selectivity towards AChE [29]. Thus, new cholinesterase inhibitors, in addition to their potential clinical importance if followed by proper pharmacological investigations, would help in defining the role of BChE in brain development, health, and ageing and would in the meantime reveal the value of both BChE and AChE inhibition as a novel strategy for the treatment of AD.

In our present study, aqueous extract of* C. acuminata* inhibited both AChE and BChE as presented in Figures 2(a) and 2(b). The inhibition of these cholinesterases could be as a result of the important phytochemicals such as caffeine and flavonoids which have already been characterized in this extract according to a previous work by Niemenak et al., 2008 [9], as shown in Figure 1. Studies have shown that caffeine is a noncompetitive inhibitor of acetylcholinesterase but not BChE according to da Silva et al., 2008 [30], as well as Pohanka and Dobes, 2013 [31]. Phenolic acids such as caffeic acid, chlorogenic acids, and catechin have been reported to be a potent inhibitor of both AChE and BChE [32, 33].

AChE is an important regulatory enzyme that controls the transmission of nerve impulses across cholinergic synapses by hydrolysing the excitatory transmitter acetylcholine (ACh) [34, 35]. BuChE, also called nonspecific cholinesterase or pseudocholinesterase, is able to act on hydrophilic and hydrophobic choline esters [36]. At this moment, the exact physiological function of BuChE is not yet clear, but it is well known that this enzyme hydrolyses a variety of xenobiotics such as aspirin, succinylcholine, heroin, and cocaine [37]. Recently, it was suggested that BuChE was found colocalised with senile plaques in the central nervous system and plays a role in the progressive beta-amyloid aggregation and in senile plaques maturation [38].

Normally, in the healthy brain AChE is predominant. However, in AD brain BChE activity rises while AChE activity remains unchanged or diminished [39]. Therefore, inhibition of both AChE and BChE by our extract is an indication that the nut could have additive and potential therapeutic benefits. Moreover, our result is in accordance with literature data that also demonstrated AChE and BChE inhibition by crude extracts from plant [40, 41].

Neurodegeneration due to oxidative stress has been implicated in the pathogenesis and progression of AD, with selective loss of cholinergic neurons in the brain being the most prominent. Studies have reported the AD brain to be under intensive oxidative stress [42] and decrease in the cholinergic neurons has been shown to promote amyloid protein deposition in the AD brain which in turn favour amyloid protein-associated oxidative stress and neurotoxicity [43]. Hence, augmenting/improvement in the body's antioxidant status through dietary means could be a practical approach through which oxidative-stress-induced neurodegeneration is controlled. In this study, incubation of rat brain tissues in the presence of 250 *μ*M FeSO_4_ caused a significant (*P* < 0.05) increase in the MDA content of the brain as presented in Figure 3(a). This finding agreed with earlier report by Butterfield and Lauderback (2002) [44] where significant increase in MDA production in rat brain was observed in the presence of Fe^2+^. The increased lipid peroxidation in the presence of Fe^2+^ could be attributed to the fact that Fe^2+^ can catalyze one-electron transfer reactions that generate reactive oxygen species, such as the reactive OH^∙^, which is formed from H_2_O_2_ through the Fenton reaction [45]. Elevated Fe^2+^ content in the brain had been linked to a host of neurodegenerative diseases and high Fe contents have been localized to degenerate regions of brains from Alzheimer's disease patients, a finding also demonstrated in animal models of the disease [46]. However, the introduction of the nut extracts inhibited MDA production in rat brain in a dose-dependent manner. This finding is consistent with our earlier report where plant extracts inhibited Fe^2+^induced lipid peroxidation in rat brain* in vitro* [44].

In addition, incubation of rat brain tissues in the presence of 7 mM sodium nitroprusside (SNP) caused a significant (*P* < 0.05) increase in the MDA production in the brain as presented in Figure 3(b). However, the extracts of the nut inhibited MDA production in rat brain in a dose-dependent manner. NO has been reported to contribute to degenerative diseases by reacting with superoxide radical (O_2_^∙−^) produced in Fenton reaction to form the powerful peroxynitrite (ONOO^−^). The ONOO^−^ can then induce lipid peroxidation, oxidation of proteins and DNA which leads to ATP-dependent PARP (poly ADP-ribose polymerase) overactivation causing neuronal ATP depletion, mitochondrial dysfunction as well as inflammation, and, ultimately, cell death [47].

Furthermore, incubating rat brain tissue homogenates in the presence of QA (a well-known excitotoxin that induces oxidative stress and damage) caused a significant (*P* < 0.05) increase in the MDA production in the brain as shown in Figure 3(c). This finding is in agreement with Butterfield and Lauderback (2002) [44] where QA caused a significant increase in the MDA content of rat brain* in vitro*. However, the nut extracts inhibited MDA production in rat brain in a dose-dependent manner. Quinolinic acid (QA) had been reported to activate neurons expressing NMDA receptors and glutamate type excitotoxicity [48]. The mechanism through which QA induces lipid peroxidation has been linked to free radical generation resulting from overstimulation of NMDA receptors. Increases in QA concentration are known to be associated with several neurodegenerative diseases including Alzheimer's disease [49]. Free radical scavengers and antioxidant enzyme inducers can protect neuronal tissue against the oxidotoxicity of QA under* in vitro* and* in vivo* conditions [50, 51].

Free radicals have an important role in pathogenesis of a wide range of diseases including AD. Antioxidants can prevent biological and chemical substances from free radical-induced oxidative damage and stress. Consequently, multipotent antioxidants have gained a great attention from scientists for their potential in treatment of many diseases [52]. Since dysregulation of metal ions such as Fe^2+^, Cu^2+^, and Zn^2+^ and consequential induction of oxidative stress have been reported to be associated with AD [46], the extracts were also decided on to screen for their antioxidant activity. Therefore, the free radical scavenging ability of the nut extracts was studied using moderately stable nitrogen-centred radical species: ABTS radical [18], DPPH free radical [20], and OH radical from the decomposition of deoxyribose [19]. Our results revealed that the nut extract scavenges free radicals in a dose-dependent manner as presented in Table 2 and Figures 4(a) and 4(b). This is an important antioxidant mechanism demonstrated by the plant and could play some part in the prevention of oxidative-stress-induced neurodegeneration.

Furthermore, the nut seed extract chelates Fe^2+^ in a dose-dependent manner. Fe chelating ability may also be one of the possible mechanisms through which antioxidants phytochemicals in nut extract prevent lipid peroxidation in tissues, and it may be by forming a complex with Fe, thus preventing the initiation of lipid peroxidation [15].

## 5. Conclusion

In conclusion, aqueous extract of kola seed (*Cola acuminata*) is rich in phenolic compounds and exhibited both anticholinesterase and antioxidant activity. This seed showed potential as functional food/or nutraceuticals in the management of neurodegenerative diseases such as Alzheimer's disease as it exhibited inhibitory activity on key enzymes (acetylcholinesterase and butyrylcholinesterase) linked to this disease. Therefore, one possible mechanism through which the nuts exert their neuroprotective properties is by inhibiting cholinesterase activities as well as preventing oxidative-stress-induced neurodegeneration. However, this is a preliminary study with possible physiological implications.

## Figures and Tables

**Table 1:** IC_50_ values for the acetylcholinesterase and butyrylcholinesterase inhibitory activities; inhibition of FeSO_4_, SNP, and quinolinic acid induced MDA production in rats brain homogenates *in vitro*; OH^∙^ and DPPH^∙^ scavenging ability as well as Fe^2+^ chelating ability.

Parameters	Values (units)
Acetylcholinesterase	14.6 ± 1.04 (*µ*g/mL)
Butyrylcholinesterase	96.2 ± 7.07 (*µ*g/mL)
FeSO_4_ induced	0.16 ± 0.12 (mg/mL)
SNP induced	0.76 ± 0.10 (mg/mL)
QA induced	0.51 ± 0.04 (mg/mL)
DPPH^∙^	2.10 ± 0.08 (mg/mL)
OH^∙^	0.97 ± 0.03 (mg/mL)
Fe chelation	1.09 ± 0.14 (mg/mL)

Values represent means ± standard deviation of triplicate readings (*n* = 3).

**Table 2:** ABTS^∙^ scavenging ability, total phenol content, and total flavonoid content of kola nut (*Cola acuminata)* seeds aqueous extract.

Parameters	Values (unit)
ABTS^∙^	2.65 ± 0.03 (mmol*·*TEAC/100 g)
Total phenol	2.78 ± 0.07 (mg*·*GAE/g)
Total flavonoid	1.75 ± 0.50 (mg*·*QUE/g)

Values represent means ± standard deviation of triplicate readings (*n* = 3).
